# Delineation of the Innate and Adaptive T-Cell Immune Outcome in the Human Host in Response to *Campylobacter jejuni* Infection

**DOI:** 10.1371/journal.pone.0015398

**Published:** 2010-11-09

**Authors:** Lindsey A. Edwards, Kiran Nistala, Dominic C. Mills, Holly N. Stephenson, Matthias Zilbauer, Brendan W. Wren, Nick Dorrell, Keith J. Lindley, Lucy R. Wedderburn, Mona Bajaj-Elliott

**Affiliations:** 1 Infectious Diseases and Microbiology, Institute of Child Health, London, United Kingdom; 2 Rheumatology, Institute of Child Health, London, United Kingdom; 3 Pathogen Molecular Biology Department, London School of Hygiene & Tropical Medicine, London, United Kingdom; 4 Paediatric Gastroenterology, Addenbrooke's Hospital, Cambridge, United Kingdom; 5 Autoimmunity and Surgery Units, Institute of Child Health, London, United Kingdom; Charité-University Medicine Berlin, Germany

## Abstract

**Background:**

*Campylobacter jejuni* is the most prevalent cause of bacterial gastroenteritis worldwide. Despite the significant health burden this infection presents, molecular understanding of *C. jejuni*-mediated disease pathogenesis remains poorly defined. Here, we report the characterisation of the early, innate immune response to *C. jejuni* using an *ex-vivo* human gut model of infection. Secondly, impact of bacterial-driven dendritic cell activation on T-cell mediated immunity was also sought.

**Methodology:**

Healthy, control paediatric terminal ileum or colonic biopsy tissue was infected with *C*. *jejuni* for 8–12 hours. Bacterial colonisation was followed by confocal microscopy and mucosal innate immune responses measured by ELISA. Marked induction of IFNγ with modest increase in IL-22 and IL-17A was noted. Increased mucosal IL-12, IL-23, IL-1β and IL-6 were indicative of a cytokine milieu that may modulate subsequent T-cell mediated immunity. *C*. *jejuni*-driven human monocyte-derived dendritic cell activation was followed by analyses of T cell immune responses utilising flow cytometry and ELISA. Significant increase in Th-17, Th-1 and Th-17/Th-1 double-positive cells and corresponding cytokines was observed. The ability of IFNγ, IL-22 and IL-17 cytokines to exert host defence via modulation of *C*. *jejuni* adhesion and invasion to intestinal epithelia was measured by standard gentamicin protection assay.

**Conclusions:**

Both innate and adaptive T cell-immunity to *C*. *jejuni* infection led to the release of IFNγ, IL-22 and IL-17A; suggesting a critical role for this cytokine triad in establishing host anti-microbial immunity during the acute and effectors phase of infection. In addition, to their known anti-microbial functions; IL-17A and IL-17F reduced the number of intracellular *C. jejuni* in intestinal epithelia, highlighting a novel aspect of how IL-17 family members may contribute to protective immunity against *C. jejuni.*

## Introduction

*Campylobacter jejuni* is one of the commonest causative agents of acute bacterial gastroenteritis worldwide [1], [2]. Infection results in clinical symptoms that can range from mild diarrhoea to severe inflammatory enteritis, infection may also precipitate Inflammatory Bowel Disease (IBD) in genetically predisposed individuals [3]. The majority of *C. jejuni* infections are self-limiting, yet intriguingly, when compared to other well-recognised enteric pathogens (e.g. *Shigella*, *Salmonella*), it is *C. jejuni* infection that precedes to a much greater extent in those who succumb to autoimmune complications such as ulcerative colitis and Guillain-Barré Syndrome (GBS) [4]. The health burden of *C*. *jejuni*-associated pathologies, in particular the significant link to morbidity in children in the developing world [5], along with emergence of antibiotic-resistant clinical isolates [6], are all factors driving current impetus for gaining further insight into *C. jejuni*-mediated disease pathogenesis [1], [2], [7].

The study of pathogenesis is currently severely hampered by the lack of a convenient animal model for infection and the fact that it is unethical to perform human studies due to the risk of volunteers developing GBS. Further, the self-limiting nature of *C*. *jejuni*-mediated gastroenteritis in the majority with few requiring hospitalisation, and the added risk of intestinal perforation severely curtails the opportunity to investigate immunity to infection *in situ*. In the present study we characterised the innate cytokine milieu generated in response to *C*. *jejuni* in an *ex-vivo* model of infection, which utilises human paediatric small intestine and colonic pinch biopsies in the co-culture system. This *in-vitro* organ culture (IVOC) system has been utilised extensively in investigating enteropathogenic *E*. *coli* (EPEC) infection [8] and work by Everest and colleagues indicates it may also be suitable for studying C. *jejuni* interactions with the human intestinal mucosa [9], [10].

Dendritic cells (DC) are critical sentinel cells that relay microbial presence either directly or indirectly (the latter *via* signals received from the overlaying epithelial lining of the gut) to naïve T cells; thus instructing the adaptive immune system to mount an appropriate response, which in a healthy host should promote successful bacterial clearance while registering memory [11]. Several studies have previously documented *C*. *jejuni*-mediated effects on murine [12] and human [13], [14] DC however, the impact of bacterial-DC cross-talk on human T cell immunity remains less clear. Here, we show that supernatants from *C*. *jejuni*-infected DC promoted significant expansion of Th-17, Th-1 and Th-17/Th-1 double-positive T cells. Collectively, our data highlights IFNγ, IL-22 and IL-17 family as critical mediators of host immunity both in the acute and in the effector phase of *C*. *jejuni* infection.

## Results

### *Ex-vivo* colonisation of human intestine by *C*. *jejuni*

**Figure 1:**
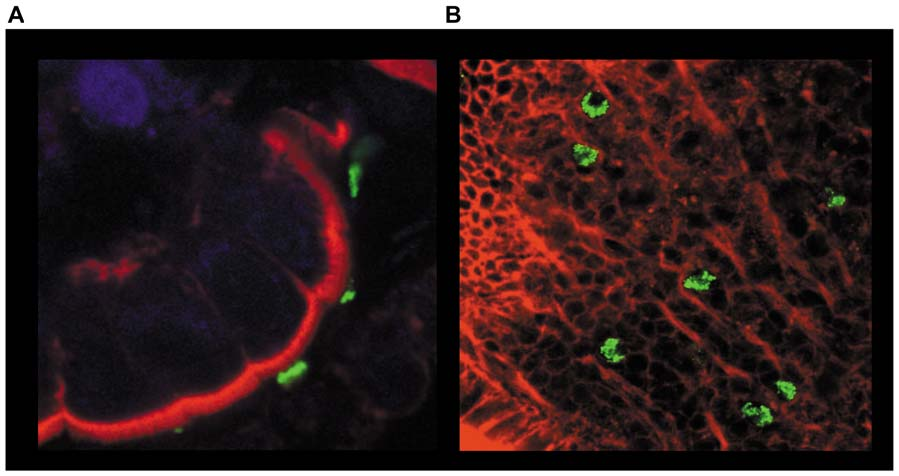
*Ex-vivo* colonisation of human intestine by *C. jejuni.* Human intestinal biopsies from the terminal ileum were co-cultured for 12 hrs with WT *C. jejuni* 11168H. Following co-culture, bacteria were localized by immuno-labelling with primary unlabelled anti-*campylobacter* antibody and secondary FITC labelled antibody (green). Actin filaments including apical brush border were visualized with rhodamine phalloidin (Red). TO-PRO blue was used to counter-stain for nuclei (blue). Whole tissue samples were examined by confocal microscopy. Transverse cross-section of the tissue (a) and an apical view (b) are shown.

Prior to investigating host mucosal immunity to *C*. *jejuni*, it was pertinent to establish if the bacteria were able to colonise the human gut explant tissue in the IVOC model of infection. Biopsies retained their 3-dimensional architecture over the 8–12 hour experimental period. A representative confocal microscopic visualisation of interaction between *C*. *jejuni* (green) and human small intestine tissue (red) is shown in Figure 1. *C*. *jejuni* were routinely found in close association with the epithelial lining (Figure 1a), with particular propensity for micro-colony formation while adhering to the small intestine (Figure 1b). Interestingly, bacterial micro-colonies were not observed during co-culture with colonic tissue (Zilbauer, M; unpublished observations).

### *Ex-vivo* release of mucosal cytokines in response to *C. jejuni* infection

**Figure 2:**
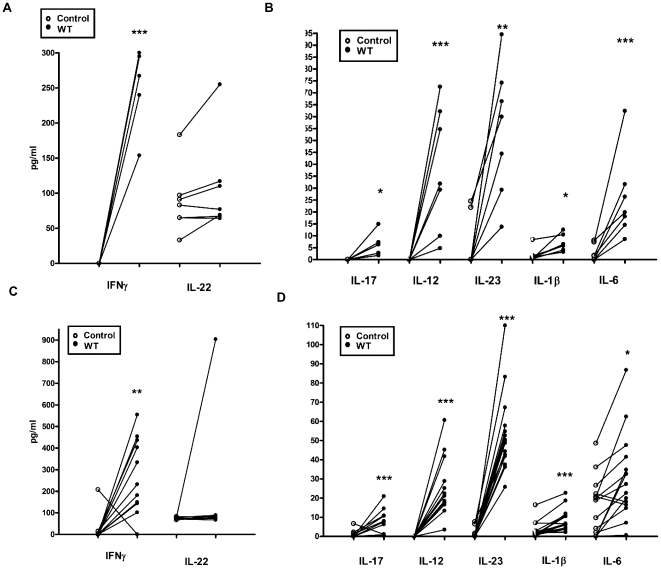
*Ex-vivo* mucosal cytokine responses to *C. jejuni* 11168H infection. Paediatric *(a)* terminal ileum and *(b)* colonic biopsy tissue were exposed to WT *C. jejuni* 11168H strain (1×10^9^/ml) bacteria for 8 hours. Post-infection IFNγ and IL-22 (a & c) and IL-17, IL-12, IL-23, IL-1β and IL-6 (b & d) protein were quantified (Data shown with median).

We aimed to determine the mucosal cytokine milieu released in response to *C*. *jejuni* with particular interest in cytokines implicated in innate defence and those involved in T cell differentiation and survival [15]. The majority of cytokines were undetectable or minimally expressed in uninfected ileal (Figure 2a & b) and colonic (Figure 2c & d) tissue during the 8 hour experimental time-period. Amongst host defence cytokines tested, IFNγ showed the most significant induction in both ileal and colonic tissue [Figure 2a & 2c; Ileal mean 251 pg/ml (SD 59.5); p = 0.0007; Colonic mean 271 pg/ml (SD 174.7); p = 0.0029]. IL-22 was secreted spontaneously by both the small and large intestinal tissue, suggesting a potential role for this cytokine in maintaining tissue homeostasis. Increase in ileal IL-22 expression was noted during infection (88 pg/ml *versus* 108 pg/ml; p = 0.948) In comparison to IFNγ and IL-22, IL-17 induction was very modest [Figure 2b & 2d; Ileal mean 6.6 pg/ml (SD 2.3); p = 0.04; Colonic mean 7.4 pg/ml (SD 6.6); p = 0.0042]. Amongst cytokines known to influence T-cell mediated immunity, IL-23 showed the most significant increase [Ileal mean 54 pg/ml (SD 27.6); p = 0.0023; Colonic mean 53.5 pg/ml (SD 20.7); p = 0.0001]. IL-12 and IL-6 increase was intermediate with IL-1β exhibiting a modest increase as noted for IL-17 protein levels.

### *C. jejuni* wild-type strains drive an IL-23/IL-12 response in monocyte-derived dendritic cells

**Figure 3:**
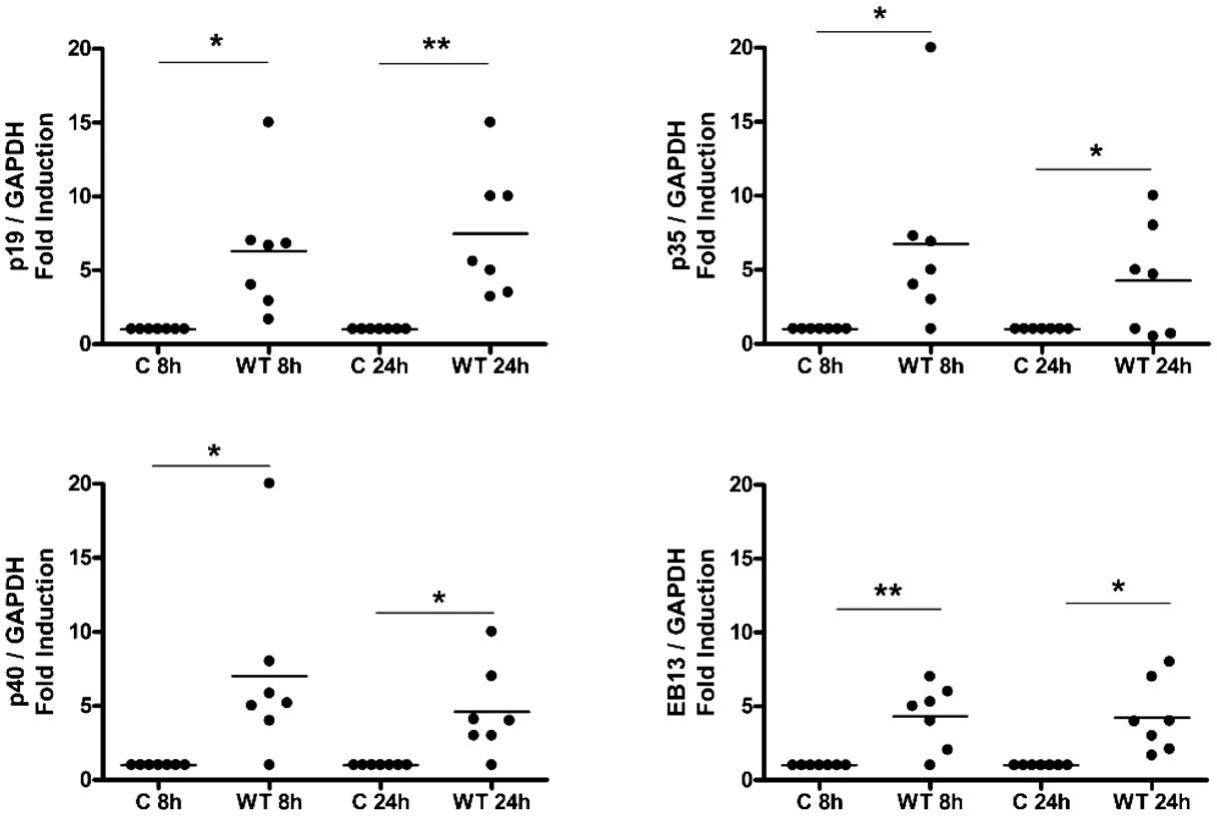
Monocyte-derived DC (DC) cytokine milieu in response to *C. jejuni* 11168H wild-type strain. DCs incubated in media alone served as Control (C) or were infected with *C. jejuni* 11168H wild-type (WT) strain (multiplicity of infection; MOI = 100). mRNA expression of the IL-12 family members (p19, p35, p40, EBI3 at 8 and 24 hours) was quantified by RT-PCR. *(a)* Gene expression was normalised to GAPDH. Variations in mRNA levels are expressed as fold induction compared to the uninfected control cells. (Median is shown). A representative gel to highlight variation in subunit expression between donors (D) is included (see Figure S1).

To decipher the molecular nature of the T cell protective immunity elicited in response to *C. jejuni* infection in humans, we first investigated the effect of *C. jejuni* infection on DC cytokine responses, with particular focus on IL-12 family members as they are critical mediators in defining the molecular nature of downstream T cell immunity. The IL-12 family members (IL-12, IL-23, IL-27 & IL-35) share subunits [16] as p35 and p40 constitute IL-12, IL-23 comprises p19 & p40, IL-27 is a hetero-dimer composed of p28 (a p35-related peptide) and Epstein-Barr virus induced gene 3 (EBI3; a p40 related protein). EBI3 can also associate with p35 to form IL-35. Further, p40 can also form biologically active homo-dimers. DCs were exposed to both wild-type (WT) *C*. *jejuni* 11168H (Figure 3) and 81–176 (data not shown) strains and expression of the IL-12 family subunits investigated by RT-PCR. In response to -*C. jejuni* infection, time-dependent differential induction of the various subunits was seen amongst the donors tested; this is highlighted in Figure S1. The majority of individuals expressed p19, p35, p40 and EBI3 in response to infection (Figure 3), however, amongst the donors tested, none showed induction of the p28 subunit (n = 7; data not shown).

**Figure 4:**
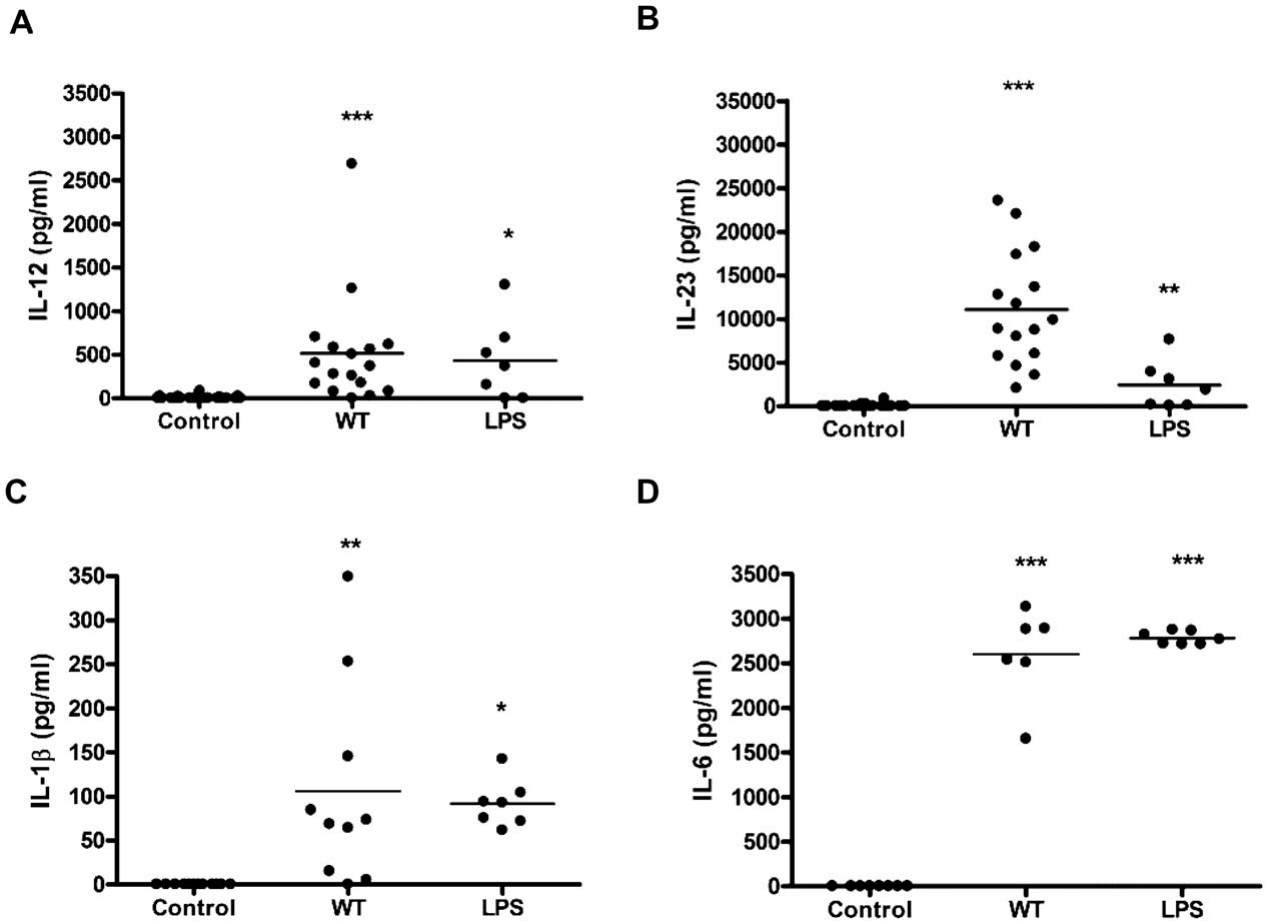
*C. jejuni* 11168H wild-type strain modulates expression of a panel of DC-derived cytokines implicated in human T cell differentiation and survival. DCs from donors were exposed to WT *C. jejuni* 11168H wild-type strain or *E. coli* LPS (10 µg/ml) for 24 hours and cytokine levels measured by ELISA. Significant increase in *(a)* IL-1β, *(b)* IL-6, *(c)* IL-12 and *(d)* IL-23 protein levels was noted (median is shown).

The cytokine responses were quantified by ELISA (Figure 4). IL-12, IL-23, IL-1β and IL-6 were undetectable in DCs exposed to medium alone. In contrast, all 4 cytokines were induced in the presence of WT *C. jejuni* infection. Interestingly, the majority of donors showed a greater propensity for IL-23 production compared to IL-12 (Figure 4). Due to the lack of commercially available antibodies against human IL-27 and IL-35 these cytokines were not quantified at protein level. Co-expression of p35 and EBI3 observed 8 hours post-infection however, does not preclude IL-35 expression.

### *C. jejuni-* infected DCs generate a cytokine milieu that favours single Th-17, Th-1 and double Th-17/Th-1 positive T cell responses

**Figure 5:**
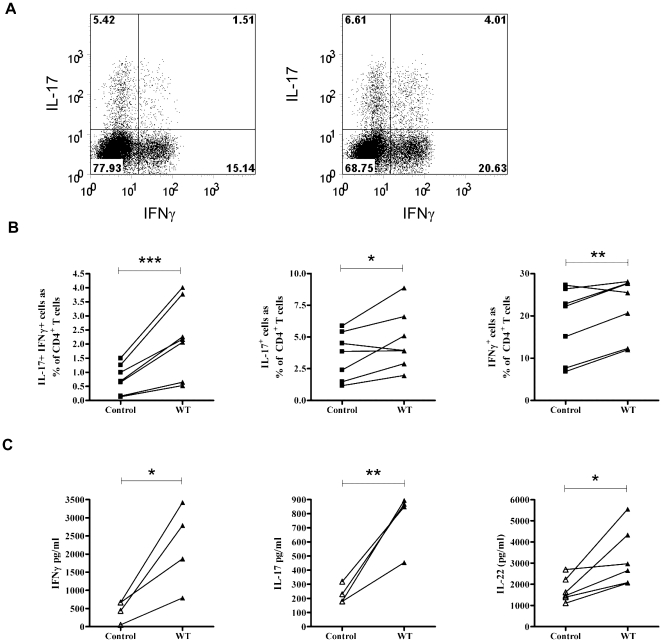
*C. jejuni* 11168H infected-DC supernatants promote expansion of Th-17/Th-1 immunity. PBMCs were enriched for CD4+CD45RO+ memory T cells and stimulated with anti-CD3, anti-CD28 coated beads for 5 days in the presence of supernatants taken from DCs cultured with medium only or with *C. jejuni* 11168H WT strain. *(a)* A representative flow cytometric plot of cultured T cells, stimulated with PMA and ionomycin for 3 hours in the presence of Brefeldin A and stained for intracellular IL-17A and IFNγ is shown. (left panel; T cells cultured in uninfected DC supernatants, right panel; T cells cultured in C. *jejuni*-infected DC supernatants). *(b)* Number of IL-17^+^IFNγ^+^ (left), IL-17^+^IFNγ^−^ (middle) and IFNγ^+^IL-17^−^ (right) cells as a percentage of CD4+ T cells, as in (a), n = 7. *(c)* T-cell derived cytokine [IFN-γ (n = 4; left), IL-17 (n = 4; middle) and IL-22 (n = 6; right),] protein quantified 5 days post-stimulation.

In the present study we focused on elucidating the impact of ‘live’ *C*. *jejuni* infection on innate and adaptive T-cell responses. The ‘live’ nature of the infection inherently prohibited us from performing standard bacterial-DC-T cell co-culture studies. DC-T cellular interactions are critical for determining T-cell proliferative capacity; the cytokine milieu generated in contrast has a major impact on T cell effector function [15]. We tested the ability of supernatants (filter-sterilised to remove live bacteria) from infected DCs (from donors shown in Figure 4) to propagate CD4+CD45RO+ T cell effector responses. Flow cytometric analysis of intracellular IFNγ and IL-17A staining showed an increase in single-positive IL-17A and IFNγ cells and a preferential increase in IL-17A/IFNγ double-positive cells. A representative plot is shown in Figure 5a. Collectively, *C. jejuni*-infected DC supernatants tested showed a significant increase in IL-17A/IFN-γ double-positive (*P* = 0.004), single IL-17A (*P* = 0.04) and single IFN-γ (*P* = 0.01) producing T cells (Figure 5b). Increase in IFN-γ and IL-17 protein was confirmed by ELISA (n = 4 donors; Figure 5c). Taken together, the data indicated that *C. jejuni*-infected DC cytokine milieu had the ability to amplify both Th17 and Th1 responses.

### T-cell derived IL-22 expression in response to *C*. *jejuni* infection

In addition to innate immune cells being a source of IL-22 [17], [18], its expression is also associated with Th-1 [19], Th-17 [20] and a distinct Th-22-cell sub-type [21], indicating widespread expression of IL-22 can occur in the intestinal mucosa. We therefore investigated if *C. jejuni* infection resulted in T-effector cells secreting IL-22. T-cell mediated IL-22 expression was indeed observed in the presence of infected DC supernatants [mean 3279 pg/ml (SD 1392) p = 0.0275; Figure 5c]. Interestingly, much more modest amounts of IL-22 (mean 18 pg/ml; data not shown) were also detected in the DC milieu itself, which did not show a significant change in response to infection.

### IL-17A and F modulate number of intracellular *C. jejuni* in intestinal epithelia

**Figure 6:**
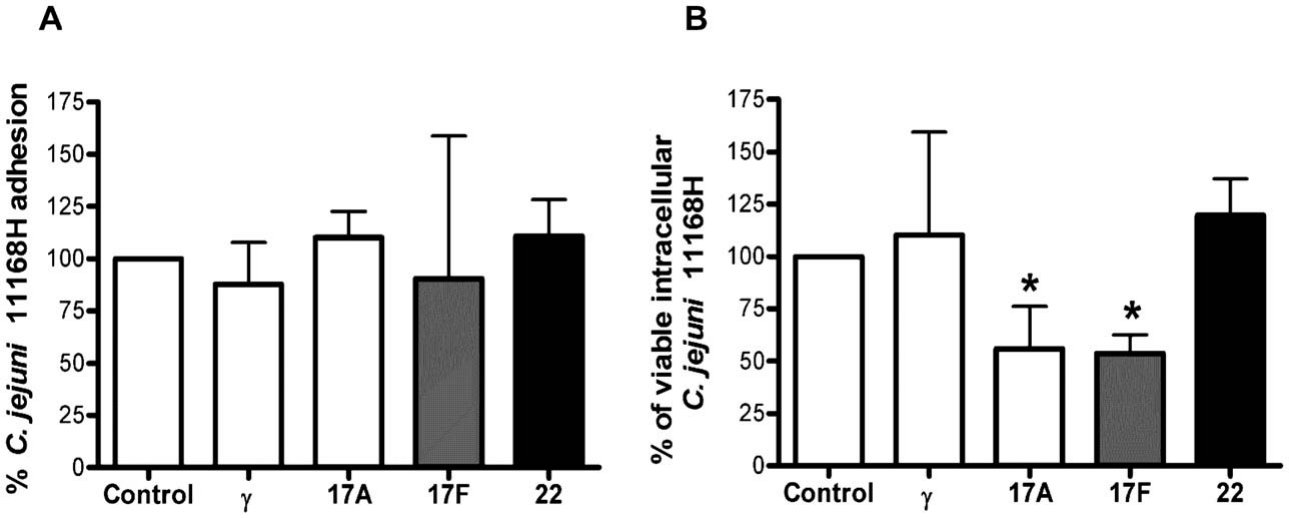
IL-17A and IL-17F reduce *C*. *jejuni* 11168H intracellular survival in intestinal epithelia. Confluent Caco-2 cells were exposed to individual cytokines for 24 hours prior to infection with *C. jejuni* 11168H WT strain (MOI = 100) for 3 hours at 37°C. Cell lysates were serially diluted and plated for total viable bacterial counts (adhesion + invasion). In parallel, another set of infected cells was exposed to 150 µg/ml gentamicin for 2 hours (to kill extracellular adhered bacteria) and lysates plated for enumeration of viable intracellular bacteria. Data represent average percentage cfu obtained in treated versus untreated cells (the latter set at 100%). Statistical analysis of 3 independent experiments performed in duplicate is shown *versus* untreated cells (Data shown as the median + range).

The generation of the mucosal, innate IFNγ/IL-22/IL-17 cytokine triad and Th-17/Th1 dual immunity with IL-22 secreting ability in response to *C. jejuni* infection suggested a critical role for these cytokines in eliciting antimicrobial immunity to *C*. *jejuni.* We hypothesised that in addition to their well-established bactericidal and tissue repair functions [15], [18] this triad may modulate *C*. *jejuni* adhesion and invasion to intestinal epithelial cells (IEC). To test this hypothesis, polarised Caco-2 cells were incubated with IFNγ, IL-17A, IL-17F or IL-22 for 24 hours (to activate cytokine-mediated IEC bactericidal cellular events) prior to exposure to *C*. *jejuni* at an MOI  = 100. 3 hours post-infection a standard bacterial adhesion and invasion assay was performed. None of the cytokines were able to modify the number of *C. jejuni* 11168H bacterial cells that adhered to IEC (Figure 6a). Pre-treatment with IFNγ or IL-22 had minimal effect on the number of viable intracellular bacteria. In contrast, IL-17A and in particular IL-17F significantly reduced the number of viable intracellular bacteria (Figure 6b).

## Discussion

Globally, morbidity and mortality in children due to diarrhoeal infectious disease(s) still remains a major challenge. Amongst known human enteropathogens, determination of the molecular pathogenesis of disease in *C. jejuni* enteritis has proven problematic for several reasons. Firstly, the possibility of generation of autoimmune antibodies due to molecular mimicry between *C. jejuni* sialylated LOS and host gangliosides as observed with GBS [4] curtails the feasibility of human volunteer studies. Secondly, the availability of a convenient small animal model that recapitulates human intestinal pathology has been limiting to date [22]–[24]. Thirdly, and most importantly, the paucity of information with regard to how the GI mucosa senses *C. jejuni* leading to protective immunity remains a major stumbling block towards our current understanding of *C. jejuni*-mediated disease pathogenesis.

In the present study we established that the IVOC model system utilising human gut explant tissue can serve as a viable model to investigate early host immunity to *C*. *jejuni*. The maintenance of tissue viability and architectural integrity over the 12 hour-co-culture period (Figure 1) added credence to the mucosal cytokine responses obtained (Figure 2). It was interesting to note that *C*. *jejuni* micro-colonies were frequently found to adhere to the small intestine; this was rarely observed with colonic tissue. Haddock and colleagues have recently reported similar findings [10]. point to note however, is that despite the lack of micro-colony formation on colonic tissue, the magnitude of cytokine response(s) elicited by the small and large intestine were found to be similar (Figure 2). Future studies must address how the GI tract senses, interacts and responds to the various guises (i.e. single cell, micro-colony and/or biofilms) that *C*. *jejuni* can adapt to [10], [25].

Analysis of mucosal immunity to C. *jejuni* allows us to hypothesise a critical role for IFNγ in particular, during the early, acute phase of infection (Figure 2a & c). Spontaneous, basal IL-22 secretion and ileal IL-22 induction post-infection are also worth noting. Both cytokines exert potent epithelial bactericidal and repair functions [18], [26]. These include enhanced production of epithelial β-defensins, which we have shown previously to be effective antimicrobials against *C*. *jejuni* [27]. IL-17A is also an agonist for β-defensin production [28]. In comparison to IFNγ and IL-22 however, IL-17A expression in response to *C*. *jejuni* was very modest (Figure 2b & d). One may speculate that IL-17A levels found maybe are sufficient in contributing to the ‘protective’ antimicrobial shield exerted by IFNγ and IL-22. The pathological effects of high IL-17 leading to ‘autoimmune reactivity’ are well-established [15], [29], [30] making tight regulation of IL-17 expression during infection a desirable option.

Currently the identity of mucosal cell type(s) that contribute to innate cytokine production during GI infection(s) in humans remains largely unknown. Many mucosal cell phenotypes including Natural Killer (NK) cells, CD8 and γδ T cells have the capacity to secrete IFNγ [31]. We found monocyte-derived DC to be low IL-22 secretors (data not shown) and this cell-type could potentially contribute to the observed mucosal IL-22 expression. A subset of NK cells; NKp46+ cells are known IL-22 producers and could also be a likely source [17], [32]. Mucosal lymphoid tissue inducer (LTi) cells can secrete both IL-22 and IL-17 and are, therefore, another potential candidate cell-type [32]. Greater availability of human reagents in the near future should greatly aid in resolving the identity of the cell-types involved.

Accumulating evidence indicates that IL-23 is a likely master regulator of mucosal immunity during GI infection and inflammation [33]. We observed mucosal (Figure 2) and DC (Figure 4) production of IL-23 in response to *C*. *jejuni.* In the majority of participants mucosal and DC-derived IL-12 and IL-23 were undetectable in the absence of the stimuli, upon infection however, most individuals showed a robust IL-23 response compared to IL-12 (Figure 4). IL-23 is a pleiotropic cytokine; it's role as a major survival factor for IL-23 receptor expressing Th-17 cells is well characterised [15]; in addition, an increasing body of emerging data indicates that IL-23 can amplify and expand Th-17 independent IL-17A and IL-22 production [29], [34]. Cecal IL-23 is a major stimulus for IL-17/IL-22 expression by γδ T cells during *Salmonella enterica* serotype Typhimurium infection in streptomycin-treated mice [35]. It would be interesting to investigate if this IL-23-mediated IL-17/IL-22 immunity occurs in human infections.

At present, the cellular source of mucosal IL-23 observed in our *ex-vivo* model system is unknown. A unique subset of CD14+ macrophages found in ‘inflamed’ human intestine has been identified as a source of IL-23 [36]. Mucosal cell-phenotypes such as the Paneth cell of the small intestine [37] and a novel innate Thy1+, ROR-γt-transcription factor expressing lymphoid cell type [33] are novel ‘potential’ candidate cells that may secrete IL-23 in response to noxious stimuli. Clearly more studies are warranted to delineate the role and contribution of the various innate cell-types to IL-23 expression during GI infection and inflammation. On the latter note, a recent epidemiological study involving 13,148 IBD patients found a significant increase in disease incidence in individuals who were exposed to *Salmonella/Campylobacter* gastroenteritis in the previous year [3]. In addition, variants of IL-23 receptor are linked to susceptibility to IBD [38]. Collectively, data suggests a role for IL-23 not only in *C*. *jejuni*-mediated host immunity, but it may also prove pivotal in modulating susceptibility to IBD post-*C*. *jejuni* gastroenteritis.

In order to gain better insight into how the DC cytokine milieu generated in response to live *C. jejuni* infection dictates cellular immunity, T cells were cultured in the presence of infected-DC supernatants obtained from various donors. A propensity for expansion of single Th-17, single Th-1 and double-positive Th17/1 cells was noted (Figure 5). Recently Lee and colleagues have shed light on the molecular nature of IL-17/IFN-γ double-positive cells [39]. Th-17 cells undergo plasticity towards a Th17/1 phenotype by up-regulating IFNγ in response to IL-12 or IL-23 in the absence of TGF-β. IL-17 expression may be completely extinguished such that Th-17 cells develop a Th-1 phenotype, though in humans, expression of the Th-17 marker CD161 is maintained on these “converted” cells [40]. This plasticity of Th-17 cells may be particularly important in mounting immunity against mucosal pathogens that can reside in different niches, i.e., remain extracellular and yet exhibit ‘invasive’ properties.

The present study implicated an innate IFNγ/IL-22/IL-17 and an adaptive Th-17/Th-1 dual response to *C. jejuni*; suggesting this cytokine triad may represent an adequate ‘antimicrobial shield’ that promotes bacterial clearance and generation of protective immunity. IL-17A and –F can homo- or hetero-dimerise and each iso-form shows distinct efficacy [41], [42]. Early neutrophil recruitment, granulocyte colony stimulatory factor, macrophage-inflammatory protein-2 and antimicrobial peptide production at the site of infection are critical functions assigned to IL-17 as lack of these responses increases systemic bacterial dissemination reducing overall survival [17], [30], [42]. Antimicrobial peptides exert their effects on microbes in multiple ways. In addition to direct bactericidal action, they can also modulate the rate of bacterial uptake by host innate cells [43]. We wished to know if IFNγ/IL-22 and IL-17-mediated activation of IEC exerted any effect on *C*. *jejuni* adhesion and invasion. IFNγ and IL-22 activation had minimal effect on *C*. *jejuni* adhesion or intracellular survival. In contrast, we clearly observed IL-17 mediated effects on the number of intracellular bacterial cells in IEC (Figure 6b). IL-17 family members can directly induce human β-defensin 2 (hBD-2) [28]; whether hBD-2 is responsible for reduced bacterial numbers or additional bactericidal signalling events promote this protection remains to be determined.

In conclusion, IFNγ, IL-22 and IL-17 family represent the cytokine triad that is likely to be protective both in the acute and effector(s) phase of *C*. *jejuni* infection. In addition to the antimicrobial spectrum exerted by the trio, IL-17A and –F may enhance mucosal immunity by modulating *C*. *jejuni* invasion and survival within IEC. A recent study showed that *C. jejuni* can survive within IEC by avoiding delivery to lysosomes [44]. This indicates that *C. jejuni* has evolved specific immune evasion strategies; manipulation of host mucosal immunity to counteract the evasion strategies is a future therapeutic challenge. Further, understanding how this ‘protective’ cytokine triad turns pathological will not only aid in better vaccine design for infectious gastroenteritis, but may also improve our understanding of how bacteria trigger and cause relapse in IBD.

## Materials and Methods

### *In-Vitro* Organ Culture (IVOC)

Intestinal biopsies were cultured as described previously [45]. 11 males and 8 females were recruited to the study (median age; 11 years). Tissue samples were microscopically examined and only intact, non-haemorrhagic tissue (orientated with mucosal surface facing upwards) was incubated in 12-well plates containing 1.0 ml of pre-warmed (37°C) IVOC medium on a rocking platform at 37°C in 95% O_2_/5% CO_2_ in a humidified incubator for 8 hours. The explant was inoculated with 1×10^9^ colony forming units (cfu) of *C. jejuni* 11168H or 81–176. The IVOC medium was replaced after 4 hours, to maintain pH and nutrient levels, and the tissue re-inoculated with bacteria as before.

### Confocal microscopy of human small bowel biopsies

Human intestinal biopsies from the terminal ileum were co-cultured, as described above, for 12 hrs with apical addition of WT *C. jejuni* 11168H. Uninfected tissue served as control (data not shown). Post-infection, tissue explants were washed 3 times in fresh IVOC media (to remove non-adherent bacteria) and fixed in 4% PFA. Tissue was permeablised by immersion in 0.2% Triton-X/PBS (Sigma, Poole, UK) for 5 min at RT. Non-specific binding was blocked by incubation in 1% BSA/PBS for 45 min. Subsequently, bacteria were localized by utilising a goat anti-*campylobacter* antibody (5 µg/ml; KPL, Maryland, USA) for 60 min at RT. After washing, tissue was incubated with FITC-conjugated rabbit anti-goat (0.5 µg/ml; Molecular probes, Eugene, Oregon, USA) secondary antibody for 30 min. Actin cytoskeleton and nuclei were stained with rhodamine phalloidin (1unit/ml in PBS/BSA; Invitrogen, Paisley, UK) for 45 min and TO-PRO blue (Molecular Probes, Euegene, Oregon, USA) for 30 min respectively. Finally, whole biopsies were mounted in Vectashield (Vector Laboratories Ltd) and visualized with a radiance 2100 confocal laser scanning microscope equipped with an argon-krypton laser and a red diode (Bio Rad Labs).

### Generation of monocyte-derived DC from Peripheral Blood Mononuclear cells (PBMC)

Monocyte-derived dendritic cells (DC) were generated as described previously [46]. Briefly, PBMC were collected from healthy donors by density centrifugation (Axis Shields, Uxbridge UK). 1×10^6^ PBMC were cultured in RPMI supplemented with 5% (v/v) human AB serum (Sigma, Poole, UK) supplemented with 100 and 50 ng/ml human recombinant GM-CSF and IL-4 (R & D, Abingdon, UK) respectively, at 37°C/5% CO_2_ for 6 days. Immature DCs were CD3-negative, CD14-low, CD19-negative, CD83-negative, CD25-negative and expressed low levels of HLA-DR, HLA-DQ, HLA-Class 1, CD40, CD86 and CD1.

### *C. jejuni*/DC co-culture

*C. jejuni* 11168H and 81–176 wild-type (WT) strains were employed. *C. jejuni* 11168H is a hyper-motile derivative of the genome sequenced NCTC11168 strain, which shows much higher colonisation levels in a chick colonisation model [47] and is thus considered the better strain to use for host-pathogen interaction studies. Strains were routinely cultured at 37°C for 24 hours on Columbia agar (Oxoid, Basingstoke, UK) plates supplemented with 7% (v/v) defibrinated horse blood (TCS Microbiology, Botolph Calydon, UK) and *Campylobacter* selective supplement (Skirrow; Oxoid) in a micro-aerobic chamber (Don Whitley Scientific Ltd, Shipley, UK) containing 85% N_2_, 5% O_2_ and 10% CO_2_. 1×10^6^ PBMC were exposed to WT *C. jejuni* at a multiplicity of infection (MOI) of 100; or to *E. coli* LPS 10 µg/ml (Sigma, Poole, UK), for 8 or 24 hours prior to analysis.

### Cytokine specific gene and protein expression

Following infection, DC or biopsies were subjected to RNA extraction utilising TRIZOL followed by cDNA synthesis (Invitrogen, Paisley, UK) and polymerase chain reaction (PCR; Bioline, London, UK). See Table S1 for specific cytokines tested.

Human IL-12 (p70), IL-23, IL-1β, IL-6, IL-17A, IFN-γ and IL-22 were measured in control, uninfected and infected DC, T cell and in biopsy supernatants using commercial ELISA kits (eBioscience, Hatfield, UK and R & D, Abingdon, UK).

### T-cell responses to *C. jejuni*-infected DC supernatants

CD4+CD45RO+ T cells were purified from healthy PBMC by magnetic bead based negative selection (Stem cell technologies, Grenoble, France). Purified T cells were cultured for 5 days with DC-derived supernatants diluted with RPMI supplemented with 5% (v/v) FCS in the presence of anti-CD3, anti-CD28 coated micro-beads (Milteyni Biotec, Surrey, UK). T cell supernatants were harvested and IFN-γ, IL-17A and IL-22 protein levels were quantified by ELISA. For intracellular cytokine analysis, T cells were harvested on day 5 and cultured for 3 hours in the presence of 50 ng/ml Phorbol Myristate Acetate (PMA), 500 ng/ml ionomycin, and 5 µg/ml Brefeldin A. Prior to antibody staining cells were first fixed in 4% (v/v) PFA in PBS and permeabilized in 0.1% (w/v) saponin. All reagents utilized were from Sigma, Poole, UK. Antibodies and wash buffer also contained 0.1% (w/v) saponin [48]. Phycoerythrin-Cyanin 7-labelled CD4, FITC-labeled IFN-γ (BD PharMingen, Oxford, UK), Alexa Fluor 647–labeled IL-17A (eBioscience, Hatfield, UK), and PE-labeled IL-22 (R&D, Abingdon, UK) were utilized. 100,000 to 200,000 events were collected with a Cyan ADP flow cytometer (Dako, Cambridgeshire, UK); for each condition, and cells were gated by their light scatter properties. Data were analyzed using FlowJo software (Tree Star, Ashland, OR).

### Gentamicin protection assay

The ability of *C. jejuni* 11168H to adhere to and invade IEC was tested on Caco-2 cells, (a human colorectal cancer cell-line) as detailed previously [49]. Caco-2 cells were exposed to 100 ng/ml IFN-γ or IL-17A, IL-17F or IL-22 (Peprotech, London, UK) for 24 hours before 1×10^8^ cfu of WT *C. jejuni* 11168H were added. Adhesion was allowed to proceed for 3 hours at 37°C. Cells were washed three times in PBS before cell lysis [2% (v/v) Triton-X100 in PBS; 15 minutes at 37°C]. Serial dilutions were plated onto blood agar plates for viable bacterial counting. For quantification of intracellular bacteria, cells were washed and incubated with 150 µg/ml gentamicin (Sigma, Poole, UK) for an additional 2 hours before proceeding with cell lysis and plating.

### Statistical Analysis

Statistical analyses were performed using GraphPad Prisim 4. Differences in gene or protein expression between control un-stimulated cells and stimulus (which will be denoted as * on reaching statistical significance) were evaluated using a two-tailed Mann-Whitney *U*-test. A p value of <0.05 was considered statistically significant (* p≤0.05 ** p≤0.01 *** p≤0.001).

### Ethics statement

Ethical approval for obtaining intestinal mucosal biopsies from patients undergoing routine endoscopic procedure was granted by the Institute of Child Health/Great Ormond Street Hospital Research Ethics Committee (06/Q0508/26). The biopsies were taken under the direction of the clinician in charge after fully informed parental consent was obtained.

Blood samples from Healthy volunteers were also obtained with informed consent and ethical approval from the Institute of Child Health/Great Ormond Street Hospital Research Ethics Committee.

## Supporting Information

Figure S1 **Monocyte-derived DC (DC) cytokine milieu in response to *C. jejuni* 11168H wild-type strain.** DCs incubated in media alone served as Control (C) or were infected with *C. jejuni* 11168H wild-type (WT) strain (multiplicity of infection; MOI = 100). mRNA expression of the IL-12 family members (p19, p35, p40, EBI3 at 8 and 24 hours) was quantified by RT-PCR. Shown is a representative gel to highlight variation in subunit expression between donors (D). (TIF)

Table S1 **Primers used in this study.** (DOC)
