# Supplementary material for: Delineation of the Innate and Adaptive T-Cell Immune Outcome in the Human Host in Response to Campylobacter jejuni Infection
Source: PLoS One. 2010 Nov 9;5(11):e15398. doi: 10.1371/journal.pone.0015398 (PMC2976761; doi:10.1371/journal.pone.0015398)
Supplement: Table S1 — Primers used in this study. (DOC) [file pone.0015398.s002.doc]

| Primer | Forward | Reverse |
| --- | --- | --- |
| IL-1α | GTC TCT GAA TCA GAA ATC CTT CTA TC | CAT GTC AAA TTT CAC TGC TTC TAT C |
| IL-1β | AAA CAG ATG AAG TGC TCC TTC CAG G | TGG AGA ACA CCA CTT GTT GCT CCA |
| IL-6 | ATG AAC TCC TTC TCC ACA AGC GC | GAA GAG CCC TCA GGC TGG ACT G |
| TNF-α | CGG GAC GTG GAG CTG GCC GAG GAG | CAC CAG CTG GTT ATC TCT CTC AGC TC |
| IL-10 | ATG CCC CAA GCT GAG AAC AAG ACC CA | TCT CAA GGG GCT GGG TCA GCT ATC CCA |
| TGF-β | GCC CTG GAC ACC AAC TAT TGC T | AGG CTC CAA ATG TAG GGG CAG G |
| p19 | TCG GCA CGA GAA CAA CTG AGG GAA C | GGA TAT GGG GAA CAT CAT TTG TAG TC |
| p35 | GCA AGA GAC CAG AGT CCC GGG AAA G | CTT CTG GAG CAT GTT GCT GAC GGC C |
| p28 | CTT TGC GGA ATC TCA CCT GCC AG | GTT GAA TCC TGC AGC CAG CAC |
| p40 | ATT GAG GTC ATG GTG GAT GCC G | GCT GGC ATT TTT GCG GCA GAT GAC C |
| EBI3 | CCT GCC TGC AGC AGA CGC CAA CG | GAG ACT CCA GTC ACT CAG TTC C |
| GAPDH | CTA CTG GCG CTG CCA AGG CTG T | CGG ATG AGG TCC ACC ACC CTG TTG |

**Table S1: Primers used in this study**
